# The Effects of Forodesine in Murine and Human Multiple Myeloma Cells

**DOI:** 10.1155/2010/131895

**Published:** 2010-10-19

**Authors:** Liesbeth Bieghs, Jo Caers, Elke De Bruyne, Els Van Valckenborgh, Fiona Higginbotham, Karin Vanderkerken, Eline Menu

**Affiliations:** ^1^Department of Hematology and Immunology, Myeloma Center Brussels, Vrije Universiteit Brussel (VUB), Laarbeeklaan 103, 1090 Brussels, Belgium; ^2^Le Departement d'Hématologie Clinique, Centre Hospitalier Universitaire de Liège, 4000 Liège, Belgium; ^3^Mundipharma International Limited, Milton Road, Cambridge CB4 0GW, UK

## Abstract

Multiple myeloma (MM) is the second most commonly diagnosed hematological malignancy, characterized by a monoclonal proliferation of malignant cells in the bone marrow. Despite recent advances in treatment strategies, MM remains incurable and new therapeutical targets are needed. Recently forodesine, a purine nucleoside phosphorylase inhibitor, was found to induce apoptosis in leukemic cells of chronic lymphocytic leukemia patients by increasing the dGTP levels. We therefore tested whether forodesine was able to inhibit proliferation and/or induce apoptosis in both murine and human MM cells through a similar pathway. We found that after 48 hours of treatment with forodesine there was a slight dGTP increase in 5T33MM and RPMI-8226 MM cells associated with partial inhibition of proliferation and a limited induction of apoptosis. When investigating the pathways leading to cell cycle arrest and apoptosis, we observed an upregulation of p27, caspase 3, and BIM. We can conclude that forodesine has some effects on MM cells but not as impressive as the known effects in leukemic cells. Forodesine might be however potentiating towards other established cytotoxic drugs in MM.

## 1. Introduction

Multiple myeloma (MM) is the second most prevalent hematological malignancy, accounting for 1% of all cancers. It is characterized by expanding malignant plasma cells in the bone marrow, secreting a monoclonal paraprotein, and inducing angiogenesis and osteolysis. Despite recent advances in therapy, MM still remains fatal with an average survival of 4 years [1]. 

Forodesine belongs to the family of purine nucleoside analogues (PNAs), but unlike PNAs it does not get incorporated into DNA/RNA [2]. Forodesine is a highly potent and specific purine nucleoside phosphorylase (PNP) inhibitor, and in the presence of dGuo, forodesine has been shown to inhibit cell proliferation by accumulating the dGTP levels in the cell [3]. Accumulated dGTP inhibits ribonucleotide reductase, thus preventing the synthesis of deoxyribonucleoside diphosphates. Depletion of deoxynucleotides eventually leads to cell cycle arrest [3, 4]. Accumulation of dGTPs has also been shown to induce oxidative stress, leading to the activation of the mitochondrial apoptotic pathway [5]. Balakrishnan et al. [6] showed that the apoptotic activity of forodesine was mediated by phosphorylation of p53 and activation of p21, while Alonso et al. [7] found that the activity of forodesine is also mediated through induction of p73 and the BH3-only member of the Bcl-2 family, BIM. BIM promotes apoptosis by modulating the interactions between the antiapoptotic and the proapoptotic members of the Bcl-2 family [8].

Forodesine has been shown to induce apoptosis *in vitro* in several malignant *T*-cell lines, such as T-cell acute lymphoblastic leukemia (*T*-ALL) [5], and B-cell lines, such as B-cell acute lymphoblastic leukemia [9] and B-cell chronic lymphocytic leukemia (B-CLL) [6, 7]. Furthermore, it is currently being tested in phase I/II clinical trials for patients with these malignancies [10–15]. It has been reported [6] that the efficacy of forodesine is partially dependent on the levels of deoxycytidine kinase (dCK) in the cells. dCK is the primary enzyme for the conversion of dGuo to dGMP, which is then converted to dGTP. As T cells have high levels of dCK, they form an attractive target for a PNP inhibitor.

To our knowledge, despite being a hematological malignancy, the effect of forodesine on MM cells has not yet been investigated. Some studies have tested the potency of the first PNAs in MM [16]. Namely, Kraut et al. [17] found that fludarabine, a nucleoside analogue of adenine, alone did not have any antimyeloma activity nor did 2-chlorodeoxyadenosine alone [18]. However, Björkstrand et al. [19] reported that in a phase II study, fludarabine increased the reduction of MM tumor cells when added to the VAD regimen (vincristine, dexamethasone, and doxorubicin).

As forodesine is more potent than the first-line PNAs and blocks proliferation through a different pathway, we tested its activity in MM cells *in vitro*. For this we used both the murine 5T33MM cells as well as the human RPMI-8226 cells. The 5T33MM model is an *in vivo* murine model that resembles the human disease closely, with development of angiogenesis. Of this model, an *in vitro* line has been developed which can grow stroma independently [20].

We examined the effects of forodesine on the dGTP levels as well as proliferation and apoptosis of the MM cells, compared to a T-ALL line, the MOLT-4 line. We also investigated the signaling pathways which lead to apoptosis and cell cycle arrest.

## 2. Material and Methods

### 2.1. 5T33MM Model

C57BL/KaLwRij mice were purchased from Harlan (Horst, The Netherlands) and used at 6 to 10 weeks of age. They were housed and maintained following the conditions approved by the Ethical Committee for Animal Experiments, VUB (license no. LA1230281). The animal ethics meet the standards required by the UKCCCR Guidelines (UKCCCR, 1998).

The 5T33MMvv cells were isolated from the BM as previously described [21]. The BM cells were suspended in supplemented serum-free medium (RPMI 1640 (GIBCO, Life Technologies, Ghent, Belgium). The 5T33MM cells with >95% viability were enriched by Lympholyte M (Cedarlane, Hornby, Canada) gradient centrifugation at 1000 g for 20 minutes and reached >80% purity (assessed by staining with anti-idiotype antibodies).

### 2.2. Cell Lines

The murine 5T33MM (MM) [20], human RPMI-8226 (MM) [22], and human MOLT-4 (T-ALL) [23] cells were maintained in RPMI-1640 medium supplemented with 10% FCS (Fetal Clone I, Hyclone, Logan, UT).

### 2.3. Reagents

Forodesine (BCX-1777) was provided by Mundipharma Research Ltd., Cambridge, England, and dGuo obtained from Sigma-Aldrich (Munich, Germany). 

### 2.4. dGTP Assay

The dGTP assay was performed according to the protocol by Bantia et al. [4]. Briefly, dGTPs from 5 × 10^6^ treated cells were extracted in methanol and dried by a TurboVap (Rotational Vacuum Concentration RVC-2-25, Harz, Germany). Primers for dGTP (5′-TTTCTTTCTTTCTTTCTTTCGGCGGTGGAGGCGG-3′; 3′-CCGCCACCTCCGCC-5′) were annealed and added, together with Klenow polymerase and ^3^H-ATP (Vitrax, CA, USA). After 30 min incubation at 37°C, cells were harvested on DE81 paper, and radioactivity was detected with a 1450 Microbeta Liquid Scintillation Counter (Wallac, Finland). Values were correlated to a known dGTP standard curve (Sigma-Aldrich).

### 2.5. BrdU Assay

BrdU (Sigma-Aldrich) was added to the cultures *in vitro* 2 hours before analysis. Cells were then fixed, permeabilized, and then stained with a FITC-anti-BrdU antibody (Roche Diagnostics, Manheim, Germany) as performed previously [24]. Flow cytometry was performed using a FACS Canto and the FACSDiva software (BD Pharmingen, Erembodegem, Belgium).

### 2.6. Annexin V/7-AAD Assay

After treatment, cells were washed and incubated with annexin V and 7-AAD (BD Pharmingen) for 15 minutes, followed by flow cytometric analysis (FACS Canto).

### 2.7. Western Blot

Cell lysates were collected as described previously [25], run over an SDS-PAGE electrophoresis, and blotted on PVDF membrane. The following antibodies were used: dCK (ab83046), Mcl-1 (ab32087) (abcam, Cambridge, UK), BIM (#2819), caspase 3 (#9665), *α*-tubulin (#2144), *β*-actin (#4967) (Cell Signaling Technology, MA, USA), c-myc (sc 764), and p27 (sc 528, Santa Cruz, CA, USA).

### 2.8. Statistical Analysis

To determine statistical significance, the Mann-Whitney test was used. *P* values <.05 were considered significant.

## 3. Results and Discussion

**Figure 1:**
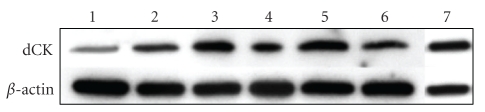
Expression of deoxycytidine kinase (dCK) in MM cells. With Western blot, the level of dCK was determined in several MM cell lines and compared to MOLT-4 cells-(1): Karpas, (2): LP-1, (3): MMS-1, (4): RPMI-8226, (5): 5T33MM, (6): 5T33MMvv, and (7): MOLT-4. *β*-actin was used as loading control; one experiment representing 3 is shown.

As the level of dCK is supposed to be a limiting factor for the activity of forodesine, we first examined the level of dCK in several MM cell lines. As can be seen in Figure 1, all MM lines express dCK, especially the 5T33MM, MMS1, and RPMI-8226 cells. Therefore, we decided to use 5T33MM and RPMI-8226 cells for further experiments.

**Figure 2:**
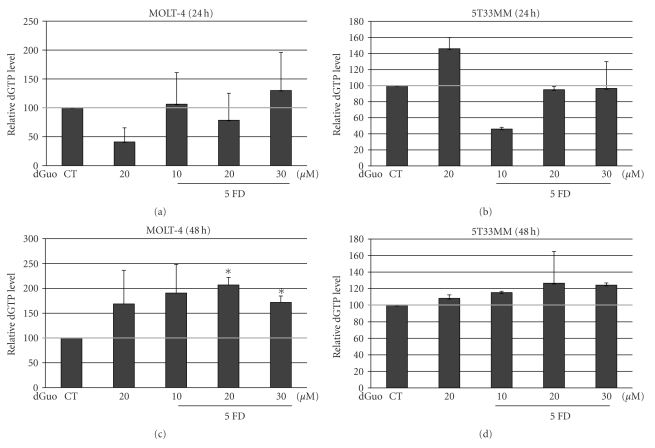
Effect of forodesine on dGTP levels in MM cells. The relative amount of dGTP was determined in MOLT-4 and 5T33MM cells, treated with 5 *μ*M forodesine and 10, 20, or 30 *μ*M dGuo at 24 hours (a, b) and 48 hours (c, d). The mean value + SD is shown of 3 independent experiments; *P < .05 compared to CT, CT = control and FD = forodesine.

We next investigated the effects of forodesine on the dGTP levels in the MM cells since the effect of forodesine is mediated by increased dGTP levels. We added an external source of dGuo to mimic *in vivo* plasma elevation of dGuo levels [6]. As it has been shown that the effects of forodesine depend on the concentration of dGuo [7], we first examined the effects of 5 *μ*M forodesine with different concentrations of dGuo on the dGTP levels in 5T33MM cells compared to MOLT-4 cells. We found that after 24 hours only limited effect could be seen (Figures 2(a) and 2(b)), but after 48 hours there was a two-fold increase in dGTP levels in the MOLT-4 cells (Figure 2(c)) and a 30% increase in the 5T33MM cells (Figure 2(d)). dGuo alone also gave an increase in dGTP levels, and this is in line with Balakrishnan et al. [6] who also found that dGuo alone could increase dGTP levels in some patient samples. In contrast, they already found a dGTP increase after 4 hours of treatment in CLL cells. This could be due to a difference in methods or a difference in cells examined.

**Figure 3:**
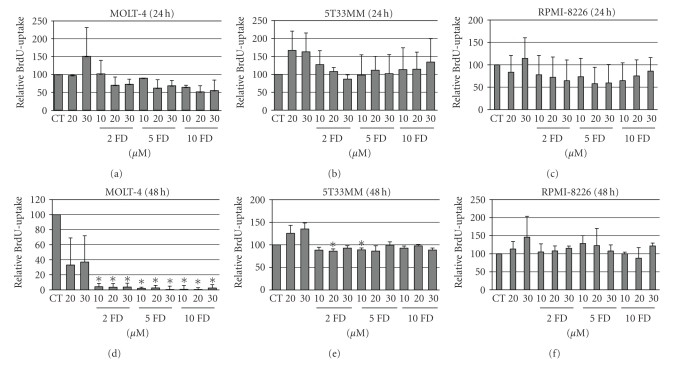
Effect of forodesine on proliferation of MM cells. The relative amount of BrdU uptake is shown in MOLT-4, 5T33MM, and RPMI-8226 cells treated with different concentrations of forodesine and dGuo at 24 hours (a, b, c) and 48 hours (d, e, f). The mean value + SD is shown of 3 independent experiments; *P < .05 compared to CT, CT = control, and FD = forodesine.

**Figure 4:**
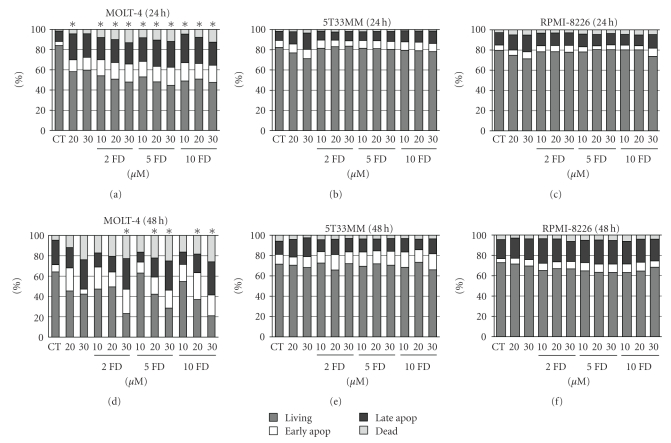
Effect of forodesine on apoptosis of MM cells. The % live, early apoptotic, late apoptotic, and dead cells are shown for MOLT-4, 5T33MM, and RPMI-8226 cells, treated with different concentrations of forodesine and dGuo at 24 hours (a, b, c) and 48 hours (d, e, f). The mean value is shown of 3 independent experiments; *P < .05 compared to CT, CT = control, and FD = forodesine.

As we observed some increase in dGTP levels in the MM cells we continued to examine the effects of forodesine on the proliferation and apoptosis of the cells. We now used different concentrations of forodesine together with different concentrations of dGuo for 24 and 48 hours. At 24 hours we saw some reduction in BrdU uptake in both MOLT-4 (Figure 3(a)) and RPMI-8226 (Figure 3(c)) cells (up to 40% reduction) but not in the 5T33MM cells (Figure 3(b)). When looking at the effects at 48 hours, we found a complete block in proliferation in the MOLT-4 cells (Figure 3(d)) and 15% reduction in the 5T33MM cells (Figure 3(e)). Examination of the number of apoptotic cells revealed that forodesine had no effect on the MM cells at 24 hours (Figures 4(b) and 4(c)), while it did reduce the percentage of living cells in the MOLT-4 cells with 40% (Figure 4(a)). At 48 hours we saw in the MOLT-4 cells a pronounced dose-dependent decrease of the living cells when treated with different concentrations of dGuo, while the concentration of forodesine made no difference (Figure 4(d)). Here also a decrease in living cells could be seen when 20 *μ*M and 30 *μ*M dGuo were added alone, correlating to the observed increase in dGTP levels. In the MM cells we observed no (5T33MM) or small (10%, RPMI-8226) effect of forodesine treatment (Figures 4(e) and 4(f)). When the 5T33MMvv cells were treated *ex vivo* with forodesine, we also found a limited effect (up to 20%, data not shown). 

**Figure 5:**
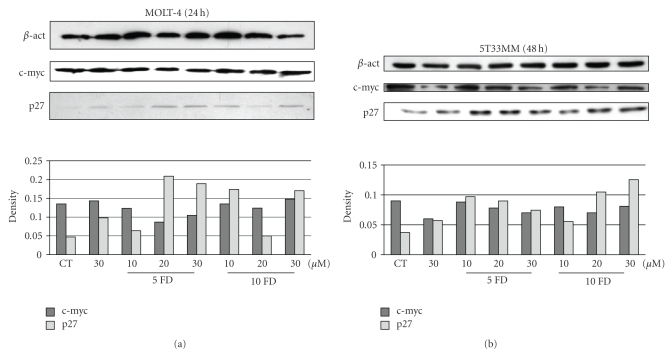
Expression of cell cycle molecules. The effects of different concentrations of forodesine and dGuo on the expression of p27 and c-myc are shown in MOLT-4 (a) and 5T33MM (b) cells. *β*-actin was used as loading control, one experiment representing 3 is shown.

We finally examined some molecular targets of forodesine in the proliferative and apoptotic pathways. To our knowledge the effects of forodesine on the different cell cycle proteins has not yet been extensively investigated. We treated 5T33MM cells for 48 hours with forodesine and MOLT-4 cells for 24 hours, so we could observe the initial effects of forodesine. We found that while forodesine had no effect on cell cycle stimulators such as cyclin D2 (data not shown) or c-myc, we did find an increase in the levels of p27, a cyclin-dependent kinase inhibitor (CKI) in both MOLT-4 cells (Figure 5(a)) as 5T33MM (5(b)), indicating that proliferation reduction is mediated by increased cell cycle inhibition. This is in line with Balakrishnan et al. [6] who found an increase in p21 in CLL cells after treatment with forodesine. Both p21 and p27 belong to the CIP/Kip family of CKIs and play a role in inhibiting all cyclins (D, E, A, and B) and Cdks (4, 6, and 2), thus inducing cell cycle arrest [26].

**Figure 6:**
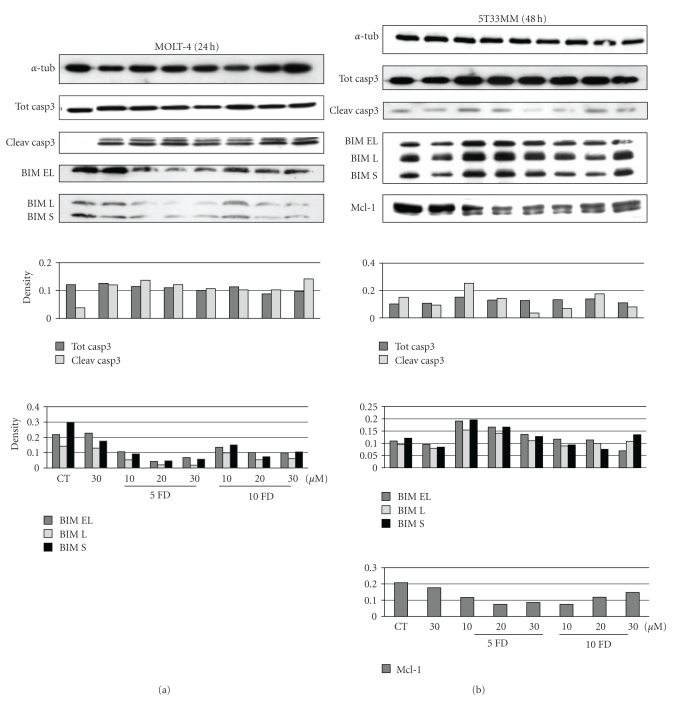
Expression of apoptosis molecules. The effect of different concentrations of forodesine and dGuo on the expression of caspase 3 cleavage, BIM and Mcl-1 are shown in MOLT-4 (a) and 5T33MM (b) cells. *β*-actin was used as loading control, one experiment representing 3 is shown.

When examining the effects of forodesine on the apoptotic pathway, we saw a clear activation of caspase 3, the effector caspase in the apoptotic pathway, in the MOLT-4 cells (Figure 6(a)), and also to a lesser extent in the 5T33MM cells (6(b)). Caspase 3 can be activated through the intrinsic or extrinsic pathway, with the intrinsic pathway initiated by the BH3-only proteins. A balance between the antiapoptotic members of the Bcl-2 family and the proapoptotic members controls the outcome of the mitochondrial apoptotic pathway [27]. Alonso et al. [7] have found that apoptosis induced by forodesine in CLL cells is mediated by an increase in proapoptotic BIM and a reduction in antiapoptotic Mcl-1, creating an imbalance in the Bcl-2 family and rendering the cells more sensitive to the proapoptotic members. We detected the same effects in the 5T33MM cells (Figure 6(b)); however, we could not see an increase in BIM expression in the MOLT-4 cells (6(a)). This discrepancy could be due to the difference in cell lineage. 

From these data it is evident that, despite having high levels of dCK, forodesine is more effective in leukemic cells than in myeloma cells. Kicska et al. [3] have also stated that dCK activity alone is insufficient to predict forodesine susceptibility. They postulated that cells resistant to anti-PNP agents may not accumulate enough dGTP to be effective because of decreased dGuo uptake and/or increased dGMP phosphatase activity. Huang et al. [28] stated that nucleoside transporters also play a critical role in the cellular transport of nucleoside analogues. They found that the equilibrative nucleoside transporters ENT1 and ENT2 were necessary for forodesine uptake and that dGuo uptake was primarily dependent on the concentrative nucleoside transports (CNTs). It could be possible that one of these is not efficient in MM cells.

Despite having limited therapeutical value in MM as a single agent, it is possible that forodesine could increase the effect of other cytotoxic therapeutics. One possible drug that might work synergistically with forodesine is the demethylation inhibitor, decitabine, as this drug also becomes activated through phosphorylation by dCK. If forodesine could potentiate the effect of existing drugs in MM, it might be beneficial to examine its effect preclinically in combination studies. 

##  Conflict of Interests

Fiona Higginbotham is an employee of Mundipharma International Limited. This work was partly supported by a sponsored research agreement from Mundipharma International Limited to K. Vanderkerken.

## List of Abbreviations

ALL: Acute lymphocytic leukemia
BIM: Bcl-2-interacting mediator of cell death
BM: Bone marrow
CLL: Chronic lymphocytic leukemia
dCK: Deoxycytidine kinase
dGTP: Deoxyguanosine triphosphate
dGuo: Deoxyguanosine
MM: Multiple myeloma
PNA: Purine nucleoside analogue
PNP: Purine nucleoside phosphorylase.
